# The comparison between the effects of pain education interventions with online and face-to-face exercise and the control group received biomedical education + standardized physical therapy in patients with chronic nonspecific neck pain during COVID-19: protocol for a parallel-group randomized controlled trial

**DOI:** 10.1186/s13063-022-06932-3

**Published:** 2022-12-20

**Authors:** Zohre Khosrokiani, Amir Letafatkar, Malihe Hadadnezhad, Yahya Sokhanguei

**Affiliations:** 1 Biomechanics and Corrective Exercise Laboratory, Faculty of Physical Education and Sport Sciences, Department of Biomechanics and Sport Injuries, Kharazmi University, Mirdamad Blvd., Hesari St, Tehran, 00982122258084 Iran; 2 University of Social Welfare and Rehabilitation Sciences, Tehran, Iran

**Keywords:** PNE, Nonspecific neck pain, Psychological factors, Pain, Disability, Function

## Abstract

**Background:**

Various psychological issues and serious health concerns during the imposed lockdown by coronavirus disease 2019 (COVID-19) have induced many changes in the treatment of patients. More effective self-management strategies through tele-rehabilitation are suggested to be applied for patients with chronic neck pain to reduce referrals to health cares and disability support through COVID-19. Also, the pain neuroscience education (PNE) approach is an educational method used by health professionals to assist patients in understanding the biology, physiology, and psychosocial factors affecting their pain experience and aligning with the cognitions and beliefs associated with pain and recurrent disability. PNE combined with tele-rehabilitation could be a new solution to encourage patients to manage their condition by themselves and increase the continuity of practice instead of face-to-face sessions.

**Objective:**

This randomized control trial (RCT) aims to investigate the effects of PNE with online and face-to-face exercise interventions, and the control group received biomedical education + standardized physical therapy on neck pain and disability, psychological factors, and function in non-traumatic chronic neck pain.

**Methods/design:**

Patients with non-traumatic chronic neck pain (patient-centered care and active involvement of patients and the public) will be recruited via flyers displayed in hospitals and universities to participate in an RCT with two experimental and one control group designed to investigate the effects of PNE with online and face-to-face exercise interventions, and the control group received biomedical education + standardized physical therapy on neck pain and disability, psychological factors, and function in non-traumatic chronic neck pain. The outcomes will be measured at baseline, after PNE, and after 3 months of an exercise intervention. All outcomes are presented as mean ± SD, and statistical significance was set at *α* level of < 0.05. The normal distribution of the variables was verified by the Kolmogorov-Smirnov test, following a descriptive analysis.

**Discussion:**

It seems that PNE plus online and face-to-face exercise interventions are appropriate educational models for the treatment of patients with neck pain during COVID-19. Also, online training seems to encourage patients to continue their treatment.

**Trial registration:**

Iranian Registry of Clinical Trials IRCT20150503022068N5. Registered on 09 September 2021

## Introduction

In 70% of patients with neck pain, there is no defined diagnosis based on the structure involved and mostly no specific cause for neck pain known as nonspecific neck pain [1]. Neck pain mainly affects adults and is associated with decreased quality of life, physical activity, and mental health [2]. Neck pain can be considered a social issue with a significant negative impact on the condition of patients, family, workplace, and the national healthcare system [3, 4].

Chronic pain is defined by the International Association for the Study of Pain (IASP) as pain beyond the normal tissue healing time (more than 12 weeks) [5]. For chronic neck pain, no underlying structural pathology is often found, and radiographic imaging findings are more age-related than patient symptoms [6]. Chronic neck pain is also described as hypersensitivity to the skin, ligaments, and muscles to the touch and passive and active neck and shoulder movements [6]. In 1983, Clifford Wolff stated that chronic pain is not only due to environmental sensitivities but “partly due to changes in spinal cord function.” [7, 8] Psychological factors (such as fear avoidance beliefs and self-efficacy) are involved in the maintenance of pain and transfer from acute to chronic pain [9].

Also, due to the coronavirus disease 2019 (COVID-19), a sudden lockdown of almost all services and activities has resulted in unexpected changes in the lifestyle of people [10] and has severely impaired their psychosocial factors, including increased anxiety, stress, and depression [11]. It has been shown that remote exercise-based treatment in Parkinson’s disease and overweight and obese individuals during the lockdown induced by COVID-19 helped patients overcome psychological issues and fitness concerns [11, 12].

Meanwhile, exercise interventions that encourage self-management are recommended for people with chronic musculoskeletal pain [13–15] and are the basic training of this approach. Due to the bio-psycho-social nature of chronic musculoskeletal pain, it seems that the educational approach based on the bio-psycho-social model is an appropriate educational model for treating these people [16, 17].

Cayrol et al. suggested that pain should be evaluated based on the factors impacting pain or daily activities [18]. Physicians may focus on behaviors such as fear of movement, passive coping strategies, and muscle activity or movement patterns that cause pain [18].

Social and psychological factors and patient preferences are important when initiating treatment planning and building therapeutic alliances [17]. During the initial evaluation, a strong therapeutic alliance can be established by introducing the concepts of pain neuroscience education (PNE) and carefully choosing words to reduce the fear of persistent pain [19]. Also, expressing problems in the patient’s own words provides more accurate information than ready-made checklists [16].

PNE gives comprehensive and adequate information on the nervous system’s response to harmful or potentially harmful stimuli; hypersensitivity to afferent neurotransmitters, such as swelling after acute injury; increased pain response in the central nervous system, hypersensitivity, or increased pain response; and neural plasticity, changes in the nervous system resulting from physical and cognitive activity [16, 19, 20].

Studies have addressed the positive effect of PNE combined with motor control training or neck/shoulder exercises on reducing the present pain, physical function, depression, anxiety, stress, quality of life, and fear avoidance in patients with musculoskeletal pain [21, 22]. However, there is evidence that shows the difference between the groups was only related to pain knowledge in the group using PNE, which could possibly lead to better coping for patients with pain in the future [23]. Louw et al. showed that no training or metaphorical method was recognized as the best to help the recovery process through the use of PNE [24].

Review studies have reported the positive effect of educational strategy based on pain neurophysiology and neurobiology on pain, disability, pain catastrophizing, and physical function [20, 25, 26].

PNE should not be used as a separate treatment method but should be combined with other treatment strategies to increase their effect (synergy) [20, 27]. This has already been investigated in a small study with positive results [28, 29] but should be considered for aggregation in further research with a large sample size and sufficient strength. Future studies should compare the effects of PNE and online exercise with PNE and face-to-face exercise and consider its cost-effectiveness [27].

Online rehabilitation reduces the treatment cost and provides 24/7 online support for home exercises [30]. Supporting exercise at home is important because self-management consistency in exercise is a common problem in exercise therapy [31, 32]. Research shows the importance of continuity of exercise at home, because it has positive effects on pain and physical function [32] and cost reduction [30].

To our knowledge, no randomized control trial (RCT) has examined the differences in the effect of PNE with online and face-to-face exercise interventions in people with chronic neck pain. Therefore, the present study aims to investigate the differences between the effect of PNE with online and face-to-face exercise interventions on neck pain and disability, psychological factors, and function in non-traumatic chronic neck pain during COVID-19. We hypothesized that PNE with online exercise intervention could be more effective than PNE with face-to-face exercise intervention on neck pain and disability, psychological factors, and function in non-traumatic chronic neck pain during COVID-19.

## Methods and study design for RCT

### Sequence generation

This study is a randomized assessor-blind controlled trial. Patients (patient-centered care and active involvement of patients and the public) with chronic non-traumatic neck pain will be recruited via flyers displayed at universities, university hospitals, and primary cares over 3 months in August, October, and November 2021in Mazandaran province, Iran. Patients interested in the study will be asked to email the researchers. All demographic data and inclusion and exclusion criteria will be gathered through online questionnaires, which will be sent in reply to patients. A physician will examine all patients meeting the criteria to ensure the selection process is carried out following the inclusion and exclusion criteria.

**Fig. 1:**
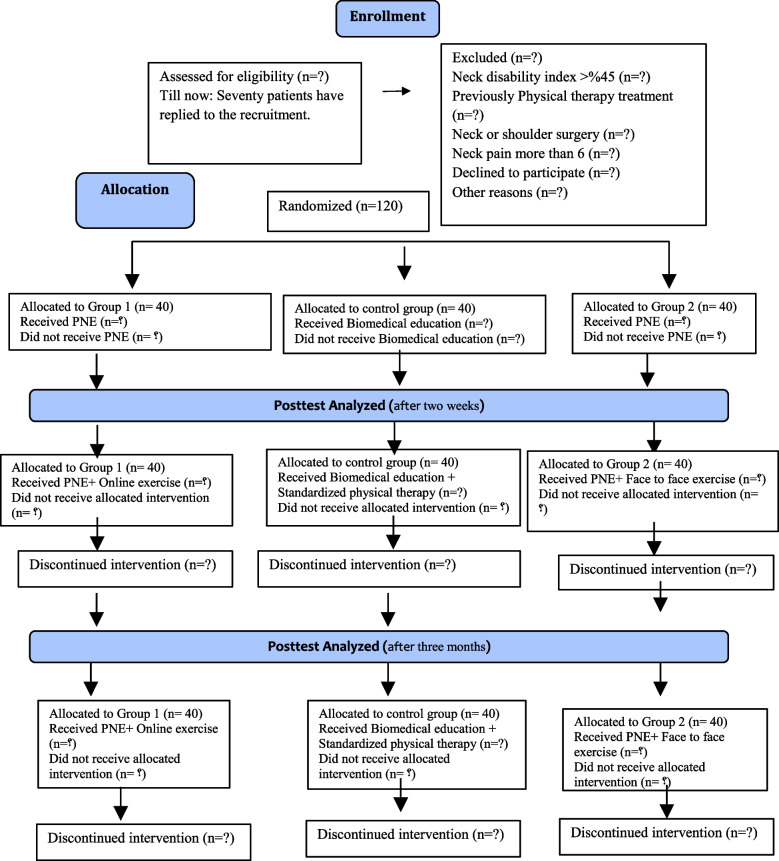
CONSORT diagram. PNE, pain neuroscience education

If the patient is interested in participating in the study, after signing the informed consent control by the researchers, the baseline data will be gathered by a blind evaluator. The patient will be informed that they can leave the study at any time. Patients in each group will be excluded from the study if they have severe pain (above 8, according to NPRS-11) or did not attend three sessions. After the initial evaluation, patients will be randomly assigned to one of the three study groups (experimental or control) (Fig. 1).

Groups will receive different treatment programs as follows:

- Group 1: PNE with an online exercise intervention once a day, 3 days a week, for up to 12 weeks
- Group 2: PNE with a face-to-face exercise intervention once a day, 3 days a week for up to 12 weeks
- Group 3: Neck biomechanical education with the recommendation to be active daily and perform a few common physiotherapy exercises once a day, 3 days a week, for up to 12 weeks

### Ethical aspects

Before starting the project, all patients will be asked to complete and signed an informed consent form. Ethics approval was obtained on 11/7/2021, from the Ethics Committee on Research at Kharazmi University, Iran (IR.KHU.REC.1400.013). Furthermore, the protocol was approved at the Iranian Registry of Clinical Trials on 2021-10-07 (IRCT20150503022068N5).

### Study participants and eligibility criteria

The patients with chronic non-traumatic neck pain who meet the following criteria will be accepted as participants in the trial: patients with chronic nonspecific and non-traumatic neck pain of at least 3 months, visual analog scale (VAS) greater than 3 from 10, both sexes range from 18 to 65, accept to take part in the research, and sign the information concept [27].

### Exclusion criteria

The patients will be excluded if they have previous history of neck or back surgery, neurological signs, rheumatoid arthritis in the past 3 years, taking part in any other therapies, and start new medication 6 weeks prior to and during participation in this trial [27].

Also, patients with chronic pain syndromes (e.g., fibromyalgia, chronic fatigue syndrome) will be excluded to obtain a homogeneous population [33].

### Randomization

Patients can discontinue the project at any time because of participant request or improving/worsening disease. However, all efforts will be made to avoid missing data. Before starting statistical analyses, the specific way to deal with missing data will be determined at a data review meeting.

### Allocation concealment mechanism

After the initial evaluation, patients will be randomly assigned to one of three study groups (PNE as an experimental group) and pain biomechanics education (control group) by selecting numbers from 1 to 120, which will be pre-prepared and packaged in sealed envelopes by an independent person. The randomization sequence will not be disclosed until patients complete their baseline assessments. The statistician and the assessor will be blind to the group allocation. Patients will not be blind to the intervention, but do not know which group will be treatment therapy. The three groups in the study will be monitored by a physiotherapist and a corrective movement instructor.

### Post-trial care

The researchers will control any adverse reports by patients through online consulting. Patients will be told to return to the research after the trial if they feel any neck pain or adverse effects.

### Interventions

All sessions of pain neuroscience training and biomedical education for patients in the experimental groups and control group, repectively, will be performed by one of the study researchers, who will be trained in seven training sessions under the supervision of a certified physiotherapist with 10 years of experience. The principles of pain neuroscience and biomedical education, the materials, goals, agenda, and activities of each session are discussed, gathered, and reviewed by two researchers.

### Interventions for experimental groups

For all patients in the two experimental groups, three sessions in 2 weeks will be held to teach PNE as well as how to perform the exercises over a period of approximately 3 months. In the first session, a power point about PNE will be presented to a group of 6 patients, the second session will be home-based online e-learning of PNE, and the third session will be a 30-min one-by-one conversation focused on the patients’ personal needs [27].

The goals of PNE include reducing the severity of pain fear, increasing patients’ awareness of pain, and conceptualizing pain. To achieve this goal, participants must understand that all pain is produced and modulated by the brain and that their pain symptoms are often related to the sensitivity of the central nervous system rather than (persistent) tissue damage [27].

The patient’s understanding of PNE will be asked in the second session. Then, in the third session, the therapist and patient will discuss these responses by relating the material to the PNE, and at the end of the PNE training, patients could put their pain in the correct perspective and feel less at risk of pain. Moreover, tend to do physical activities and not be afraid of or avoid movements.

### Interventions for the control group

Neck biomechanics is focused on clinical neck injuries and is presented by Glomsud et al. and Sokop et al. (112, 113) [34, 35]. Patients are supposed to acquire biomedical knowledge focused on neck pain. The knowledge will be causes of mechanical neck pain; anatomy; physiology and biomechanics of the bones, muscles, and joints of the spine and intervertebral discs; self-care and ergonomic managements; photos that will show the pressure inside the disc and the forces of the joints during daily activities; and postures. Lifting techniques also stretching, strengthing, and endurance exercises will be explained. The questions in the online session will also be related to the patient’s understanding and opinion about the content of the video. In the second session, the patients will be asked about their understanding of the education. In the third session, the therapist and the patient will discuss these answers by relating the questions to the educational material. Patients can also request ergonomic counseling for specific activities or situations, and lifting techniques will be practiced during this session. Patients will recommend being active daily and performing a few common physiotherapy exercises (Tables 1 and 2).

**Table 1:** Schedule of enrollment, intervention, study visits, and assessments for both study groups

Time point	Study period			
	Enrolment	Allocation	NPE, 2 weeks	Exercise intervention, 3 months
Enrollment				
Eligibility screen	*			
Informed consent	*			
Baseline assessment	*			
Allocation		*		
Intervention				
NPE			*	
Exercise intervention				*
Assessments				
NRS		*	*	*
NDI		*	*	*
DASS-21		*	*	*
PCS		*	*	*
Function		*	*	*
Adverse events			*	*

**Table 2:** Description of the exercises

Exercise	Description	Groups
A	While performing craniocervial flexion, lift the head from the foam between the neck and the mat and maintain this position for a specified period (ten repetitions, 5–6 repetitions, one set).	1, 2, 3
B	In the supine position, apply gentle pressure to the foam between the neck and the mat (ten repetitions, 5–6 repetitions, one set).	1, 2, 3
C	In the supine position, apply gentle pressure to an elastic band, which is placed in front of the forehead and held with both hands (ten repetitions, 5–6 repetitions, one set).	1, 2
D	In the supine position, apply gentle pressure to an elastic band, which is placed behind the head and held with both hands (ten repetitions, 5–6 repetitions, one set).	1, 2
E	In the supine position, hold an elastic band with both hands with the shoulders in the neutral position and the elbows at an angle of ninety, while simultaneously moving the left and right shoulders outward (ten repetitions of 5–6 s, one set).	1, 2
F	Hold the shoulders abducted while holding an elastic band (ten repetitions of 5–6 s, one set).	
G	While performing craniocervical flexion, lift the head from the foam between the neck and the mat and holds this position for a specified period (10 s, one set).	1, 2, 3
H	In the supine position, place an elastic band on the cervical spine from behind and hold it with both hands, applying gentle pressure to it at different levels of the cervical spine (one repetition of 5–6 s per level, one set).	1, 2
I	While performing craniocervical flexion in the supine position, perform flexion, rotation, and extension of the lower part of the cervical spine (ten repetitions of 10 s, one set).	1, 2, 3
J	In the supine position, hold an elastic band with both hands with the shoulders in the neutral position and the elbows at an angle of ninety, while simultaneously moving the left and right shoulders outward (ten repetitions, 10 s, one set).	1, 2
K	In the supine position, flex shoulders with an elastic band resistance (ten repetitions per shoulder).	1, 2
L	Perform craniocervicral flexion in the four kneeling positions while holding the lumbar, thoracic, and cervical spine in a neutral position (ten repetitions of 10 s, one set).	1, 2, 3
M	On four kneeling while performing craniocervical flexion, extend the neck and thorax, retract the shoulders, and flex the elbows (ten 10-s repetitions, one set).	
N	While performing craniocervical flexion, lift the head from the foam between the neck and the mat and maintain this position for a specified period (ten repetitions of 10 s, two sets).	1, 2, 3
O	In the supine position, place an elastic band on the cervical spine from behind and hold it with both hands, applying gentle pressure to it at different levels of the cervical spine (10 s in each level, one set).	1, 2
P	External movements of rotation and diagonal of the shoulders with an elastic band (ten repetitions, two sets).	1, 2
Q	In the four kneeling positions, in the correct alignment of the lumbar spine, neck, and thorax, perform craniocervical flexion (two sets of ten in Yes mode and two sets of ten in No mode).	1, 2
R	In the supine position on a large pilates ball, while performing craniocerebral flexion, extend the thorax and retract the shoulders (ten 10-s repetitions, two sets).	1, 2
S	In the supine position, raise the head for a certain period of time while holding the craniocervical flexion (ten repetitions of 10 s, two sets).	1, 2, 3
T	In the supine position, the person performs rotation and diagonal movements with an elastic band (ten repetitions, two sets).	
U	In the four kneeling positions, while performing the correct alignment of the cervical, thoracic, and lumbar spines, the person performs craniocerebral flexion (ten repetitions, two sets).	
V	In the prone position on a pilates ball, the cranioservital flexion is performed by extending the head with an elastic band resistance (five repetitions per level, holding for 5 to 6 s).	

### Exercise intervention for experimental groups

These exercises will be performed to increase the endurance and strength of the flexor and deep extensor muscles of the neck [36] and the stabilizing muscles of the scapula [37]. These exercises will be presented to the patients during three face-to-face sessions (the first session will discuss only PNE in the experimental groups and neck biomechanics training in the control group). Each exercise is performed from one to four sets of ten repetitions. The number of exercises for each muscle group is added from one exercise in the fourth session to four in the last session. Exercise time will increase from 15 min in the first session to 30 min in the last session. Online group patients are supported and accounted for 24/7 online by the researchers. Also, offline group patients will be answered in person only once a week, and more if necessary. Each group will continue their training interventions for 12 weeks (Tale 2, 3). Borg rating of perceived exertion of 11–13 was chosen to progress the intensity of the training from light to somewhat hard over the 12-week exercise program [38].

All patients will be going to limit their weekly exercise to the study program. The prescheduled exercises will be flexible according to each individual’s progression and limitations (Appendix).

### Outcome variables

All variables will be assessed initially (before intervention), after PNE (after 2 weeks), and after exercise intervention (after 3 months). The flow chart can be seen in Fig. 1. After filling in the questionnaires by the patients, they will be asked about taking any painkillers and psychological medication. The completed questionnaires will be handed over to a skilled psychiatrist who will be blind to assigning patients to study groups. The psychiatrist will consider if it is necessary for any of the patients to take any psychological medication.

Personal information about potential and enrolled participants will be collected, shared, and maintained to protect confidentiality before, during, and after the trial.

### Initial socio-demographic and clinical characteristics of patients

1. Sex (both males and females)
2. Age (between 18 and 65)
3. Neck pain intensity in accordance with VAS
4. Neck pain history (month)
5. Level of studies (no studies, basic education, secondary school, university studies)
6. Employment situation (student, active, active in temporary disability, unemployed, domestic occupation)
7. Marital status (single, widowed, couple, separated or divorced)
8. Weight (kg), height, and body mass index (weight [kg]/height [m^2^])
9. Smoking status (at present, never, before)

### Primary outcome measurements

The primary outcome will be measured as between- and within-group differences in the Numerical Pain Rating Scale (NPRS) at the second and third assessments.

#### Numerical Pain Rating Scale (NPRS)

The scale is 11 points for pain reporting. This is the most common pain reporting scale. The patient will choose a number according to the rule (0–10) that best indicates the severity of his pain. The number zero indicates no pain and 10 indicates the most severe or worst pain. (There are different types of numbers from 0 to 10 that one can choose). The scales are categorized as follows: painless (0), mild pain (1–3), moderate pain (4–6), and severe pain (7–10), but these categories do not necessarily reflect the patient’s meanings and for any assessments are poor. These categories may be used to set goals for the outcome of the intervention. NPRS can be done orally (by phone) or graphically. The validity of the NPRS has been shown in patients with rheumatism and other chronic pain conditions (pain > 6 months), ICC has been reported to be 0.95–0.86.40. The MDC reported from the 11-point NPRS is 0.45 [39].

### Secondary outcome measurements

#### Neck Disability Index (NDI)

The Neck Disability Index (NDI) is a questionnaire [40] consisting of ten sections that show the effect of neck pain on a person’s daily activities. The ten sections include determining the severity of pain, activities such as personal care, studying, headaches, concentration, work, driving, sleep, lifting, recreation, and entertainment.

In each part, the patient receives a score between 0 and 5, with 0 indicating no problem and a score of 5 indicating the most problem. The total score obtained from the neck pain and disability questionnaire is between 0 and 50, which is in five levels: 0–4 without disability, 5–14 low disability, 15–24 moderate disability, 25–34 severe disability, and 35–50 disability. Its reliability and internal coherence are well reported [40, 41]. In cases where the activities mentioned in the questionnaire are not in the person’s daily schedule, the scores are adjusted and calculated based on the percentage of the total score. In this trial, the disability index, a score between 15 and 30 or 30 to 60% of the total adjusted score, was considered [40]. It has been shown that this questionnaire has high validity (correlation with Magill pain questionnaire = 0.70) and excellent reliability (ICC = 0.89) [42]. Its reliability and internal consistency are well reported (ICC = 0.80) (109) [43]. The least clinically significant difference (MCD) in people with chronic nonspecific neck pain is 20% [44].

#### Pain Catastrophic Scale (PCS)

The Pain Catastrophic Scale (PCS) is used to assess catastrophic pain perceptions. PCS consists of 13 items scored on a 5-point scale. The total score is between 0 and 52. The higher scores indicate a more severe pain disaster [45]. The ICC has been reported to be 0.87–0.87 for PCS [46].

#### Depression, Anxiety, and Stress Scale-21 (DASS-21)

DASS-21 applies psychological distress components (depression, anxiety, stress) on a 21-item ranking scale with scales for each item 0 “does not apply to me at all” to 3 “very” or most of the time [47, 48]. This questionnaire has good structural validity and provides improved normative data and rating scales to help describe the clinical severity (mild/moderate/severe/very severe) for each scale. Scores greater than 20 (depression), more than 14 (anxiety), and more than 25 (stress) indicate a “severe” rating [47, 48]. Reliability for depression anxiety and stress is 0.96, 0.89, and stress 0.95, respectively [49, 50].

#### Deep Neck Flexor Endurance Test

Neck flexor muscle endurance will be measured using Grimmer’s deep neck flexor endurance test [51]. The person was placed in the supine position on the examination bed with his hands on the bed next to his body and puts the soles of his feet on the bed. He was asked to bend his chin towards his neck, keep his head about 2.5 cm away from the bed, and then maintain this position as much as possible [51]. The length of time the patient was able to maintain this position will be recorded. The test shows the activation and isometric endurance of the deep cervical flexors as well as their interaction with the superficial cervical flexors. If the position is not fully maintained, the test will be stopped. The validity of this test has been confirmed [52]. No MDC seems to have been reported for this test.

### Adherence

It is believed if patients’ autonomy, perceived competence, and relatedness are satisfied, patients’ participation in treatment will be more autonomous and less controlled. This will occur when the patient will be given valued benefits of the treatment by his/her therapist or healthcare and then willing to participate. Also, the patient’s participation in the treatment could be enhanced through a satisfying relationship with his/her therapist [53]. Through this process, the researchers will try their best for patients to feel that the treatment is potentially the best thing for their health and is consistent with their goals [54].

### Statistical analysis

The study was designed to identify a 2-point difference in pain intensity (30%) assessed by the NPRS across groups, with an expected standard deviation of 2.5 points [55]. For the global perceived effect, there is a difference of 2 points on the scale, with 11 points ranging from − 5 to + 5 [39]. The other specifications were an alpha of 5% and a power of 80%, with a total of 288 patients. However, assuming a 20% drop in follow-up, we will enroll 360 individuals (88 patients per group). G*Power was used to calculate the sample size (GPower 3.0.10, University of Kiel, Germany). The intention-to-treat protocol was followed for post-treatment analysis. See the CONSORT diagram for details.

## Discussion

For several years, treating patients with chronic pain has been more evaluated and has been given more attention due to the burden of prevalence and cost that the patient, his/her family, and society have to handle [56]. This RCT aims to investigate the differences between the effects of PNE with online and face-to-face exercise interventions on neck pain and disability, psychological factors, and function in 120 patients with non-traumatic chronic neck pain during the lockdown imposed by COVID-19. The outcomes of this RCT may help patients with neck pain living in lockdown to overcome their pain through webs which is more cost-effective and time-consuming than face-to-face treatment.

Tele-rehabilitation is home-based, professionally guided training sessions accessed via telecommunication devices such as video calls or pre-recorded classes. It is mostly used nowadays because of COVID-19, which allows treatment of patients without the risk of infection for neither the patient nor the health professionals [57].

Technological adjunctive techniques, such as remote health, have been used successfully in the rehabilitation of conditions such as stroke [58] and orthopedic surgery [59] and promise to provide psychological treatment. Such approaches have not yet been used in chronic neck pain exercises; however, evidence suggests that inactive treatment is not necessary for neck pain conditions. Instead, active treatment approaches that improve patients’ self-efficacy are preferred because of the strong evidence that they can be presented in more innovatively ways than traditional face-to-face sessions.

Also, the results of a clinical trial by Traeger et al. (2018) have shown that the PNE approach can reduce possible referrals for health care over 3 months (but not 12 months) compared to the control group [60]. Given the heavy financial burden of chronic musculoskeletal pain on health care, PNE has been shown to reduce intra-group health care consumption in a sample of musculoskeletal pain and can therefore be considered a cost-effective intervention [61, 62].

In summary, the protocol of this RCT is inexpensive and needs minimal equipment. However, to be sure about the persistence of the results, it is suggested that this approach could be further investigated in a follow-up of 12 months.

### Trial status

The trial is currently underway. It is planned for the study to complete by May 1, 2022.

## Appendix

Table 3

**Table 3:** Common exercises used in three groups

Week	Exercise	Number of sets and repetitions	Rest between each repetition	Rest between each set	Groups
Week 1	A, B, C, D, E	10 repetitions of 5–6 s, 1 set	10 s	20 s	1, 2
Week 2	B, C, D, E, F, G	Exercises B, C, D, and E, 10 repetitions of 10 s, 1 set; exercise F, 10 repetitions of 5–6 s, 1 set	10 s	20 s	1, 2
Week 3	D, E, F, G, H, I	Exercises B, C, D, E, F, and I, 109 repetitions of 10 s, 1 set; exercise G, 1 repetition of 5–6 s per level, 1 set	10 s	20 s	1, 2
Week 4	D, E, F, G, H, I, J	Exercises D, E, F, H, and I, 10 repetitions of 10 s, 2 sets; exercise J, 10 repetitions of 10 s, 1 set	10 s	20 s	1, 2
Week 5	E, F, G, H, I, J, K	Exercises E, F, H, I, and J, 10 repetitions of 10 s, 2 sets; exercise K, 10 repetitions for each shoulder	10 s	20 s	1, 2
Week 6	F, G, H, I, J, K, L	Exercises F, G, H, I, and J, 10 repetitions of 10 s, 2 sets; exercise K, 12 repetitions for each shoulder; exercise L, 10 repetitions of ten 1–2 s, 1 set	10 s	20 s	1, 2
Week 7	G, H, I, J, K, L, M	Exercise G H, I, J, and L, 10 repetitions of 10 s, 2 sets; exercise K, 12 repetitions for each shoulder; exercise M, 10 repetitions of 10 s, 1 set	10 s	20 s	1, 2
Week 8	G, H, I, J, K, L, M, N	Exercises G, H, I, J, L, and M, 10 repetitions of 10 s, 2 sets; exercise K, 12 repetitions for each shoulder; exercise N, 10 repetitions of 10 s, 1 set	10 s	20 s	1, 2
Week 9	I, J, K, L, M, N, O, P	Exercises I, J, L, M, and N, 10 repetitions of 10 s, 2 sets; exercise K, 12 repetitions for each shoulder; exercise O, 10 s per level, 1 set; exercise P, 10 repetitions, 2 sets	10 s	20 s	1, 2
Week 10	J, K, L, M, N, O, P, Q, R	Exercises J, L, M, and R, 10 repetitions of 10 s, 2 sets; exercise K, 12 repetitions for each shoulder; exercise O, 10 s per level, 1 set; exercises P and Q, 10 repetitions, 2 sets	10 s	20 s	1, 2
Week 11	K, L, M, N, O, P, Q, R, S, T	Exercises L, M, R, and S, 10 repetitions of 10 s, 1 set; exercise K, 12 repetitions for each shoulder; exercise O, 10 s per level, 1 set; exercises P, Q, and T 10 repetitions, 2 sets	10 s	20 s	1, 2
Week 12	M, N, O,P, Q, R, S, T, U, V	Exercises M, R, and S, 10 repetitions of 10 s, 2 sets; exercise K, 10 repetitions for each shoulder; exercise O, 10 s per level, 1 set; exercises P, Q, T, and U, 10 repetitions, 2 sets; exercise V, hold 5 repetitions in each level, 5–6 s	10 s	20 s	1, 2

Common exercises in all three groups: the number of repetitions and the maintenance time of the mentioned exercises are the maximum required, but can be changed according to the individual’s ability. It should be noted that the subjects did a warm-up program for 5 min before each training session and a 5-min cooling program after each training session

## Abbreviations

COVID-19: Coronavirus disease 2019
PNE: Pain neuroscience education
RCT: Randomized control trial
IASP: International Association for the Study of Pain
VAS: Visual analog scale
NPRS: Neck Pain Rating Scale
NDI: Neck Disability Index
PCS: Pain Catastrophic Scale
DASS-21: Depression, Anxiety, and Stress Scale-21
